# Pelargonidin improves functional recovery and attenuates neuropathic pain following spinal cord injury in rats: relevance to its neuroprotective, antioxidant, and anti-inflammatory effects

**DOI:** 10.3389/fphar.2025.1547187

**Published:** 2025-03-24

**Authors:** Leila Kooshki, Sajad Fakhri, Fatemeh Abbaszadeh, Amir Kiani, Mohammad Hosein Farzaei, Ehsan Mohammadi-Noori, Javier Echeverría

**Affiliations:** ^1^ Student Research Committee, Kermanshah University of Medical Sciences, Kermanshah, Iran; ^2^ Pharmaceutical Sciences Research Center, Health Institute, Kermanshah University of Medical Sciences, Kermanshah, Iran; ^3^ Neurobiology Research Center, Institute of Neuroscience and Cognition, Shahid Beheshti University of Medical Sciences, Tehran, Iran; ^4^ Regenerative Medicine Research Center, Health Technology Institute, Kermanshah University of Medical Sciences, Kermanshah, Iran; ^5^ Departamento de Ciencias del Ambiente, Facultad de Química y Biología, Universidad de Santiago de Chile, Santiago, Chile

**Keywords:** spinal cord injury, pelargonidin, motor activity, neuropathic pain, neuroinflammation, neuroprotection, oxidative stress

## Abstract

**Background:**

Spinal cord injury (SCI) significantly impairs individuals’ sensorimotor functions, hindering daily activities. Current therapeutic options often demonstrate limited efficacy and lead to undesirable side effects. Emerging research highlights the potential of anthocyanins, especially pelargonidin, which possess neuroprotective, anti-inflammatory, and antioxidant properties beneficial for neurological conditions.

**Purpose:**

This study sought to explore the impact of intrathecal administration of pelargonidin on the recovery of sensory-motor functions and associated disorders in a rat model of SCI through neuroprotective effects and regulating inflammatory/oxidative stress mediators.

**Materials and methods:**

In total, 35 male Wistar rats were divided into five groups: sham, SCI, and three treatment groups receiving different intrathecal concentrations of pelargonidin (1, 2, and 4 mM) once on day 0 after surgery/injury. Weight changes were assessed and behavioral analyses were done, including hot plate tests, acetone drop tests, von Frey tests, inclined plane tests, as well as Basso, Beattie, and Bresnahan (BBB) scores, weekly up to day 28 post-injury. On day 28, serum levels of nitrite, catalase, and glutathione as well as matrix metalloproteinase (MMP) assays and histological evaluations were done.

**Results and discussion:**

Pelargonidin significantly attenuated neuropathic pain, improved motor performance, and reduced weight loss in rats with SCI. Biochemical assays demonstrated increased serum catalase/glutathione level, and MMP2 activity, while decreased serum nitrite level and MMP9 activity. Histological analyses showed an enhancement in the number of motor neurons in the ventral horn of the spinal cord after treatment with pelargonidin, highlighting its neuroprotective and neurogenic effects.

**Conclusion:**

Pelargonidin makes substantial therapeutic benefits following SCI by accelerating sensorimotor recovery. This effect is likely due to its strong antioxidant, anti-inflammatory, and neuroprotective properties.

## 1 Introduction

Spinal cord injury (SCI) is a multifaceted medical condition that profoundly affects individuals’ healthcare systems. This condition results from spinal cord damage and can be triggered by both traumatic and non-traumatic events (Diop and Epstein, 2024). The World Health Organization (WHO) reports that the global incidence of SCI varies between 8 and 246 cases per million people each year (Kumar et al., 2018). Beyond the immediate physical consequences, SCI is associated with various secondary complications encompassing a cascade of biological events such as inflammation, oxidative stress, and neuronal apoptosis. These secondary processes can exacerbate initial damages, leading to further loss of function and complicating recovery efforts (Anjum et al., 2020).

Currently, available treatments for SCI are limited, primarily focusing on symptom management and the prevention of secondary complications. Surgical interventions can stabilize the spine or decompress neural structures, but they do not guarantee functional recovery. Pharmacological approaches, such as analgesics and anti-inflammatory medications, help manage pain but often come with adverse effects that complicate the clinical picture (Alvi et al., 2024). Notably, there is no Food and Drug Administration (FDA)-approved medication against SCI. While some studies suggested that high doses of methylprednisolone may offer neuroprotective benefits, many investigations indicated that this treatment may be ineffective in reducing neurological impairment and could even worsen spinal tissue injury, leading to severe side effects (Evaniew et al., 2015; Jeong et al., 2021). Consequently, there is a pressing need for innovative therapeutic strategies to enhance recovery and improve functional outcomes for individuals with SCI.

In recent years, there has been increasing interest in the potential of natural compounds, particularly those derived from plants, to provide neuroprotective benefits and improve recovery after SCI (Coyoy-Salgado et al., 2019; Abbaszadeh et al., 2020). Among these phytochemicals, anthocyanins are water-soluble pigments found in a variety of fruits, vegetables, and plants that have garnered attention for their therapeutic properties (Mohammadi Pour et al., 2019; S; Jaiswal et al., 2020; Chen et al., 2024). As a notable anthocyanin, pelargonidin is commonly found in strawberries, raspberries, and other red fruits. Research suggests that pelargonidin exhibits potent anti-inflammatory, antioxidant, and neuroprotective effects (Roghani et al., 2010; Sohanaki et al., 2016; Duarte et al., 2018), making it a promising candidate for addressing the oxidative stress and inflammation associated with SCI.

Oxidative stress significantly contributes to SCI by increasing reactive oxygen species (ROS) production, which worsens neuronal damage and triggers secondary injury mechanisms like apoptosis (programmed cell death) (Yu et al., 2023). As another dysregulated cross-talk pathway to oxidative stress and apoptosis, inflammation also plays a critical role in SCI complications leading to further complications (Chan, 2008). Therefore, the use of antioxidant/anti-inflammatory pelargonidin could provide a dual benefit in promoting sensory-motor recovery and protection of neural tissues.

This study sought to investigate the effects of pelargonidin on motor function recovery and neuropathic pain in a rat model of SCI through antioxidant, anti-inflammatory, and neuroprotective effects.

## 2 Material and methods

### 2.1 Animals

In total, 35 adult male Wistar rats, weighing 230–250 g, were kept in a light/dark (12-h) cycle. This study was conducted following the guidelines of the animal care committee at Kermanshah University of Medical Sciences (IR.KUMS.AEC.1401.017). The rats were assigned to five groups at random: Sham (laminectomy with no injury then receiving 10 μL intrathecal distilled water), SCI (laminectomy + injury, then receiving 10 μL intrathecal distilled water), and three treatment groups receiving three doses (1, 2, and 4 mM) of pelargonidin (purchased from Sigma-Aldrich). Thirty minutes following the compression injury, the SCI group received an intrathecal injection of distilled water as a vehicle for sham and SCI groups, while the three pelargonidin groups were given 10 μL of pelargonidin at concentrations of 1, 2, and 4 mM.

#### 2.1.1 Spinal cord injury

The rats were given a combination of ketamine/xylazine (80/10 mg/kg, intraperitoneal, purchased from Alfasan, Netherlands) to achieve deep anesthesia. A laminectomy was then carried out at the thoracic 8-9 (T8-T9) level a Micro Rongeur (Fine Science Tools, United States). Following the surgery, an aneurysm clamp made a force of 90 g (Aesculap, Tuttlingen, Germany) for 1 minute to induce severe SCI in the SCI and pelargonidin groups. After the surgical procedure, the tissue and skin were sutured. Daily administrations of cefazolin (40 mg/kg) and 2 mL of normal saline were provided, along with manual bladder massage, until normal bladder function was regained.

#### 2.1.2 Intrathecal drug injection

For the intrathecal injection, a 25-G needle connected to a Hamilton syringe was utilized. The needle was inserted at a 45° angle into the subarachnoid space between the lumbar 6 (L6) and L5 vertebrae. The confirmation of needle placement in the subarachnoid space was based on the immediate lateral tail movement observed upon penetrating the ligamentum flavum. Once the needle was correctly positioned, a dosage of either pelargonidin or distilled water (10 μL) was gradually injected over 10 s. To prevent the backflow of medication, the syringe was left in place for an additional 10 s after the injection (Mestre et al., 1994).

### 2.2 Behavioral test

The animals underwent behavioral testing on day 0 and on days 7, 14, 21, and 28 post-surgeries. All behavioral assessments were conducted in a blinded manner.

#### 2.2.1 Assessment of motor performance

The Basso, Beattie, and Bresnahan (BBB) locomotor rating scale was employed to evaluate locomotor recovery in rats following SCI. The scale ranges from 0 to 21, with 0 indicating no hindlimb movement and 21 representing normal, coordinated locomotion. The final score was calculated by averaging the scores of both hind paws (Abbaszadeh et al., 2018; Fakhri et al., 2022c).

In addition, the ability of rats to bear their body weight and maintain a standing position on an inclined surface serves as a critical metric for evaluating their recovery post-SCI. For this assessment, a wooden plane measuring 60 × 40 cm was employed, which could be inclined at angles from 0° (horizontal) to 60°. The maximum angle at which the rats were able to sustain their position for 5 s was recorded (Fakhri et al., 2018; Zhang et al., 2023).

#### 2.2.2 Assessment of neuropathic pain; cold allodynia, heat hyperalgesia, and mechanical pain

Acetone was utilized to assess pain sensitivity and behavioral responses to cold stimuli, called cold allodynia. During the test, 0.1 mL of acetone was applied from a distance of 2 cm to assess the rats’ reactions, which were categorized and scored based on their hind paw withdrawal behavior. (0) no paw withdrawal, (1) the rat exhibited a startled reaction but did not withdraw its paw, (2) the rat slightly withdrew its paw in response to the cold sensation, (3) the rat withdrew its paw for an extended period, indicating discomfort, (4) the rat licked or flinched its paw, demonstrating a heightened sensitivity to the cold stimulus (Allchorne et al., 2005).

In the hot plate test, rats were positioned on a heated surface with a temperature of 50 ± 2°C. The duration until they displayed signs of discomfort, such as licking or biting their hind limbs, was measured and documented as the latency of paw withdrawal (PWL) (Xu et al., 2024).

Mechanical allodynia was evaluated using the von Frey test, which involved applying filaments exerting forces of 10, 15, 26, 60, and 100 g to the plantar surface of the rat’s paw. The application of each filament was repeated five times, with a brief 5-s interval separating each application. A positive response was defined as any nocifensive behavior, including hindpaw withdrawal, flinching, licking, or biting. To determine the allodynia threshold, the criterion required was a minimum of three positive responses out of the five applications (Alimoradian et al., 2024).

#### 2.2.3 Weight change measurement

The weights of the rats were recorded every week. Weight changes for each group were calculated as follows:

Weight difference=(weight on days 7,14,21,and 28 post‐surgery ‐ weight on day 0)

### 2.3 Zymography

On day 28 of the study, a gelatin zymography assay was performed to analyze the activity of MMP2 and MMP9. This technique utilized 7.5% SDS-PAGE gels that were copolymerized with 0.1% gelatin. Samples were collected from the aorta of rats, loaded onto the gels, and subjected to electrophoresis at a constant voltage of 150 V, which facilitates the separation of proteins based on size. The gels were washed with a buffer containing Triton X-100 (purchased from ACROS) and Tris-HCl (Tris Base purchased from Merck Company, Germany) to remove the SDS (purchased from Merck Company, Germany), allowing the gelatin to retain its structure for enzyme activity. The gels were then incubated for 18 h at 37°C in a buffer containing 10 mM CaCl_2_ (purchased from Merck Co, Germany), 0.15 M NaCl, and 0.02% NaN_3_ in 50 mM Tris-HCl (pH 7.5), which provided the necessary conditions for the MMPs to degrade the gelatin. At this stage, the gels were stained using Coomassie Blue (Sigma-Aldrich), a dye that binds to proteins, creating a blue background. The areas where enzymatic activity occurred, where the MMPs had degraded the gelatin, appeared as clear bands against this blue background. These clear bands indicate the presence and activity of MMP2 and MMP9. Finally, ImageJ software was used to quantify band intensity (Bagheri Bavandpouri et al., 2024).

### 2.4 Biochemical test

#### 2.4.1 Catalase and glutathione activity assay

Catalase activity was determined using the Aebi method (Aebi, 1984). 20 μL of rat serum was incubated with 100 μL of a 65 mM hydrogen peroxide solution at 25°C for 4 min. The reaction was stopped with 100 μL of ammonium molybdate and measured at 405 nm using an ELISA reader.

Glutathione activity was assessed via Ellman’s method (Eyer and Podhradský, 1986). In this procedure, 20 μL of rat serum was added to the wells, followed by the addition of 50 μL of phosphate buffer at pH 7. Then, 40 μL of dithiobisnitrobenzoic acid (DTNB) was included, and the plate was incubated at 37°C for 10 min. The absorbance was subsequently read at 412 nm using an ELISA reader. Finally, the percentage difference in absorbance for both catalase and glutathione was calculated between the other groups and sham groups. 

#### 2.4.2 Nitrite assay

Nitrite levels were assessed using the Griess reaction assay. To prepare the serum samples, protein removal was achieved by adding zinc sulfate, followed by centrifugation of the samples obtained from rats on day 28. The supernatant was then mixed with vanadium chloride solution at a 1:1 ratio. A Griess solution containing 2% sulfanilamide and 0.1% diamide dihydrochloride was added, and the samples were incubated at 37°C for 30 min. The resulting color was measured at 540 nm using a spectrophotometer, and nitrite concentration in the samples was determined by comparing the absorbance to that of a standard reference (Sun et al., 2003).

### 2.5 Histological analysis

On day 28, the animals were perfused with normal saline and 4% paraformaldehyde. Subsequently, a 1-cm segment of spinal cord tissue centered on the injury was excised. The spinal cord tissue was embedded in paraffin, sliced into 7-μm sections, and stained with hematoxylin and eosin (H&E). For lesion size, 4X magnification was used to calculate the ratio of damaged tissue to the total cross-sectional area from images taken across all study groups, expressing this ratio as a percentage relative to the sham group. This method enabled a detailed comparison of tissue damage among the experimental groups. For neuron counting, we captured images from the ventral horn region of the spinal cord tissue sections from each sample at 40X magnification. Using ImageJ software, we determined the average number of neurons in each section of the ventral horn across different groups and compared these numbers (Li et al., 2014).

### 2.6 Statistical analysis

GraphPad Prism, Version 8.0, was utilized for data analysis. The results are shown as mean ± SD. To compare the groups, a repeated measures one-way or two-way analysis of variance ANOVA was conducted, followed by Tukey’s or Bonferroni’s *post hoc* tests as appropriate. A significance threshold of *p* < 0.05 was established for all statistical evaluations.

## 3 Results

### 3.1 Behavioral result

#### 3.1.1 Pelargonidin significantly enhanced motor function in rats with SCI

**FIGURE 1:**
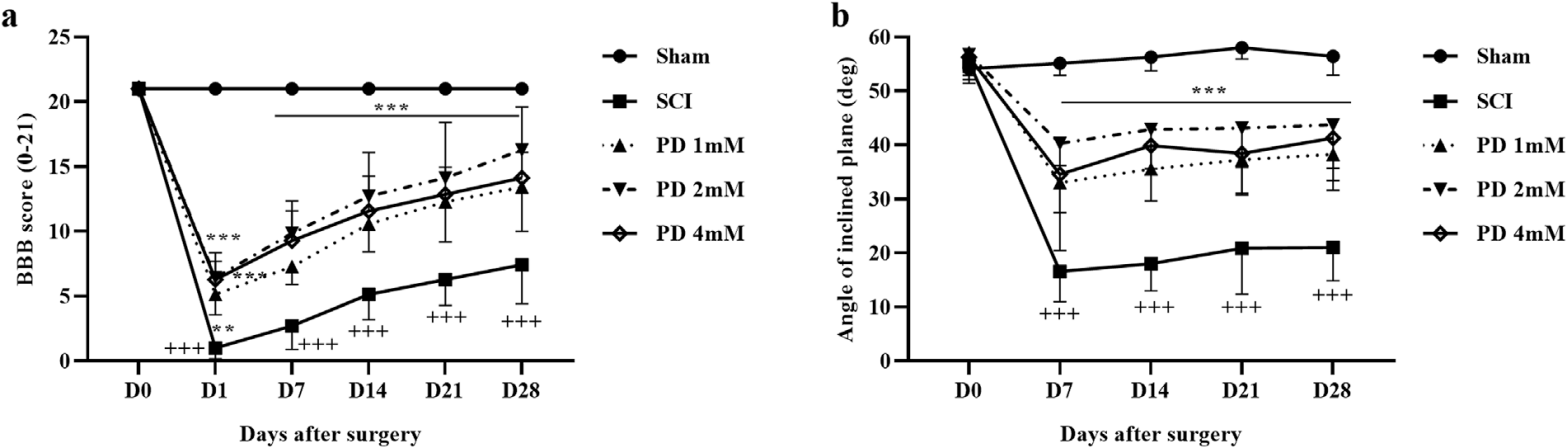
The impact of pelargonidin on motor activity after SCI. BBB score **(a)**, and inclined-plane **(b)** test. Data are expressed as mean ± SD (*n* = 7). The statistical analysis was done using repeated measures of two-way ANOVA. ^+++^ *p* < 0.001 vs. sham group; ^**^ *p* < 0.01, ^***^ *p* < 0.001 vs. SCI group. SCI: spinal cord injury; PD: pelargonidin.

We evaluated the motor performance of rats following SCI using the BBB scale and the inclined plane test. The sham group, which underwent a laminectomy without injury, consistently scored 21 on the BBB scale, indicating no functional deficits. In contrast, SCI rats exhibited significantly lower scores of 2 on the first day post-injury, which remained substantially below the sham group’s average score of approximately 8 until day 28 (*p* < 0.001). Importantly, treatment with pelargonidin at various doses led to significant improvements in motor function starting from day 1, compared to the SCI group (*p* < 0.001, Figure 1a).

In terms of balance, the sham group was able to maintain their balance on an incline of up to 55°. However, the SCI group exhibited a significant decrease in their average angle of balance, dropping by about 25° (*p* < 0.001) compared to the sham group. Conversely, rats treated with pelargonidin showed considerable enhancements in balance from the first week, achieving angles between 30° and 40° (*p* < 0.001, Figure 1b).

#### 3.1.2 Pelargonidin significantly alleviated neuropathic pain in rats with SCI

**FIGURE 2:**
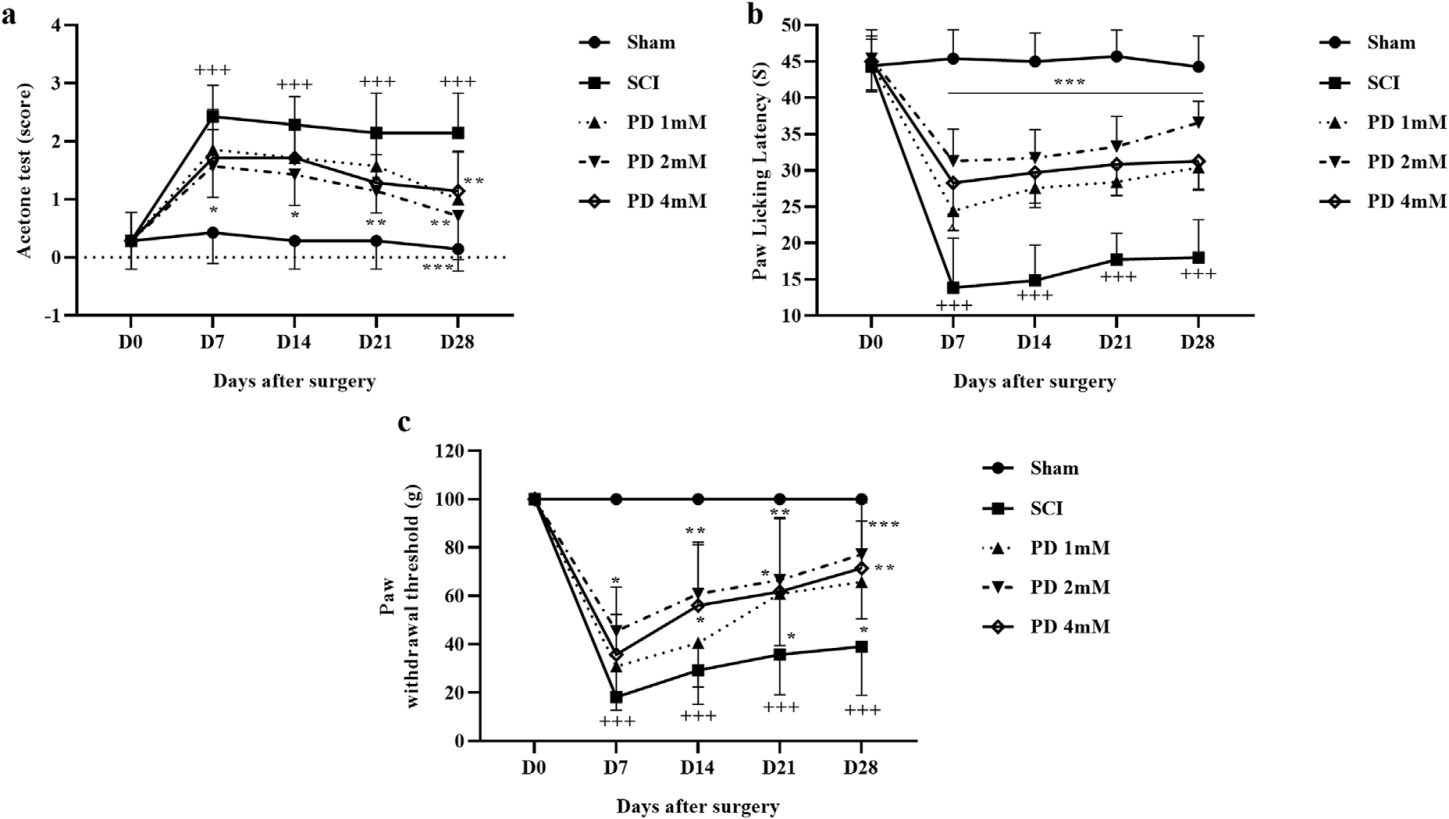
The impact of pelargonidin on neuropathic pain after SCI. Acetone **(a)**, hot plate **(b)**, and von Frey **(c)** test. Data are expressed as mean ± SD (*n* = 7). The statistical analysis was done using repeated measures of two-way ANOVA. ^+++^ *p* < 0.001 vs. sham group; ^*^ *p* < 0.05, ^**^ *p* < 0.01, ^***^ *p* < 0.001 vs. SCI group. SCI: spinal cord injury; PD: pelargonidin.

Throughout the 4 weeks, the sham group maintained typical sensitivity to cold pain stimuli, whereas the SCI group demonstrated marked hypersensitivity (*p* < 0.001). Treatment with various doses of pelargonidin, especially at 2 mM, resulted in a notable improvement in response to cold stimulation from the first week (*p* < 0.05, Figure 2a).

In the current study and during the hot plate test, rats in the sham group displayed a stable paw-licking latency across all testing days and no significant changes in pain perception or response. When rats experienced SCI, there was a marked decrease in their response threshold to thermal stimuli starting from day 7 of the study, meaning they were more sensitive to the thermal stimulus (*p* < 0.001). In the following, treatment with different doses of pelargonidin led to a significant decrease in sensitivity to thermal stimuli as evidenced by an increased paw-licking latency (*p* < 0.001, Figure 2b).

Also, the sham group demonstrated consistent responses to mechanical stimuli throughout the 28-day follow-up period, and they did not exhibit any significant changes in pain perception or withdrawal behavior. In contrast, rats in SCI groups showed a significant increase in sensitivity to mechanical stimuli, as indicated by a decrease in the paw withdrawal threshold (*p* < 0.001). Notably, administration of pelargonidin, particularly at a dose of 2 mM, effectively alleviated the sensitivity of the response to the stimulus and symptoms of mechanical allodynia beginning from day 7 post-treatment (*p* < 0.05, Figure 2c).

#### 3.1.3 Pelargonidin effectively regulated weight changes in rats with SCI

**FIGURE 3:**
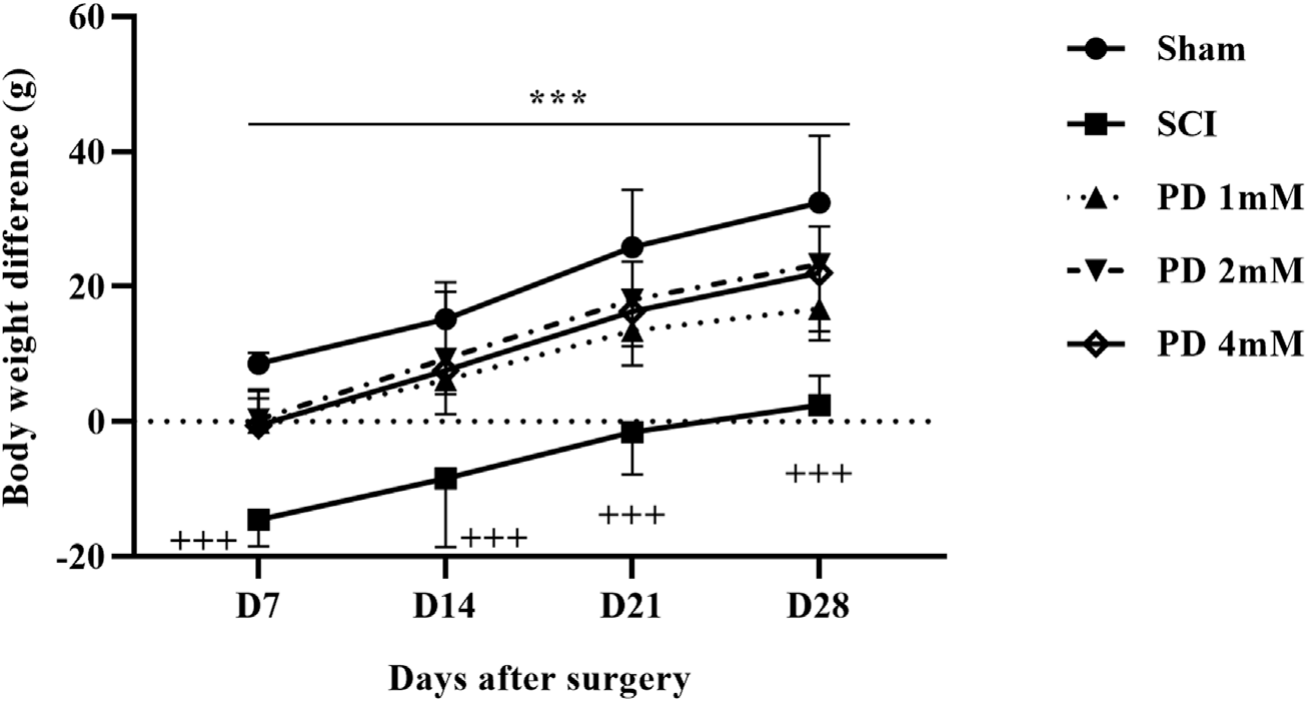
Effects of pelargonidin on body weight changes after SCI. Data are expressed as mean ± SD (*n* = 7). The statistical analysis was done using repeated measures of two-way ANOVA. ^+++^ *p* < 0.001 vs. sham group; ^***^ *p* < 0.05 vs. SCI group. SCI: spinal cord injury; PD: pelargonidin.

Rats in the shame group displayed a normal weight gain pattern, indicating their physiological condition remained unchanged. In contrast, the SCI group experienced notable weight loss, underscoring the considerable effects of the injury on their overall health and metabolic function. Notably, the administration of different doses of pelargonidin resulted in a significant enhancement in weight gain for the injured animals (*p* < 0.001, Figure 3).

### 3.2 Pelargonidin modulated oxidative stress markers in rats with SCI

**FIGURE 4:**
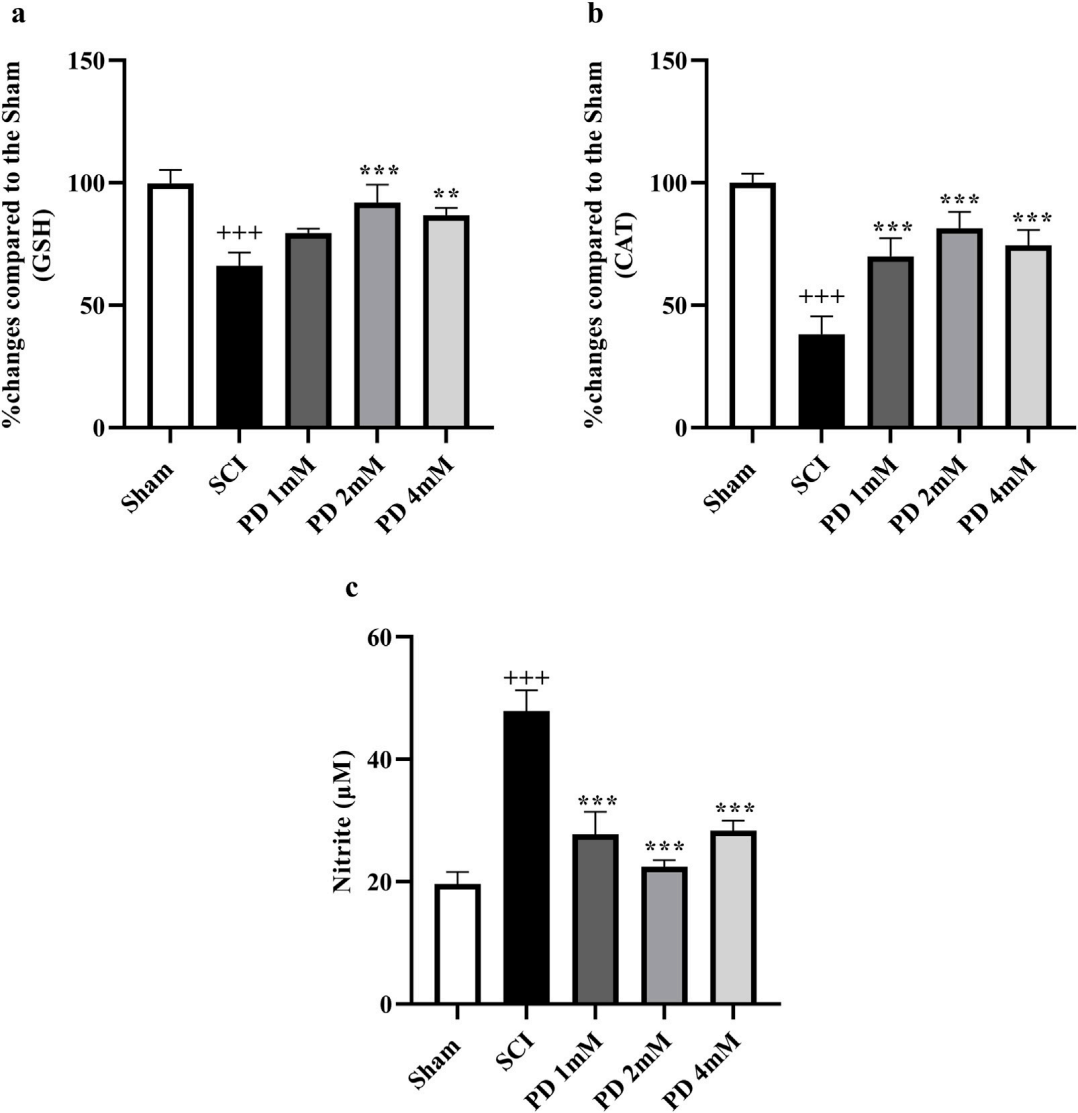
Effect of pelargonidin on changes of oxidative stress after SCI. Percentage of activity changes of glutathione **(a)**, catalase **(b)**, and level of nitrite **(c)**. Data are expressed as mean ± SD. The statistical analysis was done using repeated measures of one-way ANOVA. ^+++^ *p* < 0.001 vs. sham group; ^**^ *p* < 0.001, ^***^ *p* < 0.001 vs. SCI group. SCI: spinal cord injury; PD: pelargonidin.

Our study findings revealed that compared to the sham group, SCI significantly reduced serum levels of glutathione (Figure 4a) and catalase (Figure 4b), key antioxidants, indicating increased oxidative stress (*p* < 0.001). Treatment with pelargonidin effectively restored these antioxidant levels (*p* < 0.05). In addition to the changes in antioxidant levels, SCI was associated with an increase in serum nitrite levels (Figure 4c), which can be indicative of heightened nitrite production and further oxidative stress. However, treatment with pelargonidin also resulted in a significant reduction of these elevated nitrite levels (*p* < 0.001).

### 3.3 Pelargonidin effectively modulated MMP levels in rats with SCI

**FIGURE 5:**
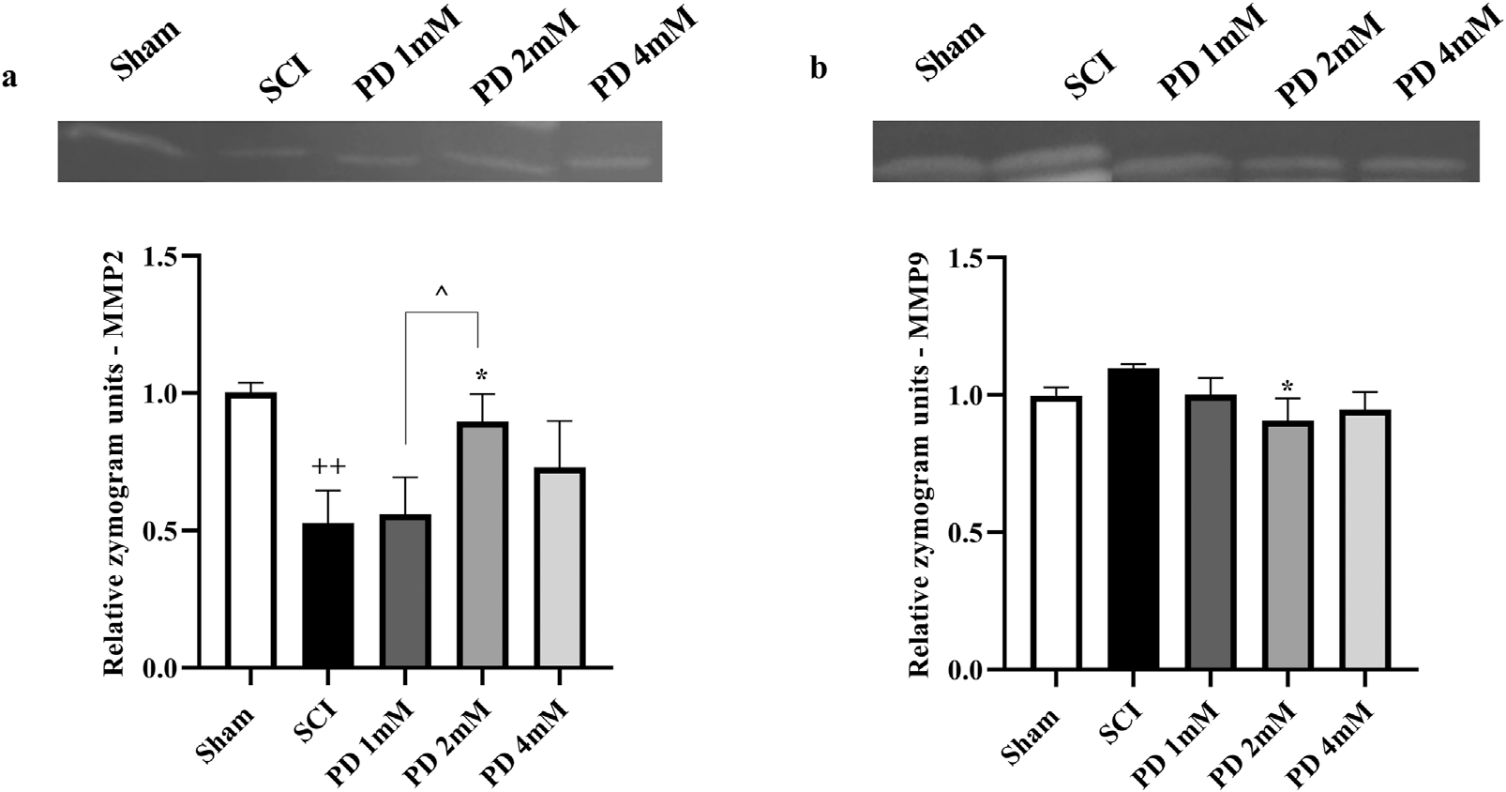
Effects of pelargonidin on MMPs level after SCI. MMP2 **(a)** and MMP9 **(b)**. Data are expressed as mean ± SD. The statistical analysis was done using repeated measures of one-way ANOVA. ^+^ *p* < 0.05, ^+++^ *p* < 0.001 vs. sham group; ^*^ *p* < 0.05, ^**^ *p* < 0.01 vs. SCI group; ^*p* < 0.05 vs. PD 2 mM group. SCI: spinal cord injury; PD: pelargonidin. MMP: Matrix metalloproteinase.

After SCI, the analysis showed a significant reduction in MMP2 levels (*p* < 0.001, Figure 5a), indicating a potential loss of its protective anti-inflammatory function. Concurrently, MMP9 levels were significantly elevated (*p* < 0.001, Figure 5b), which could suggest a heightened inflammatory response and contribute to secondary injury processes. However, treatment with varying doses of pelargonidin, particularly at a concentration of 2 mM, resulted in a remarkable reversal of these trends. Pelargonidin administration not only restored MMP2 levels (*p* < 0.05) to more favorable values, enhancing its anti-inflammatory effect but also significantly lowered MMP9 levels (*p* < 0.05).

### 3.4 Pelargonidin mitigated histological alterations in rats with SCI

**FIGURE 6:**
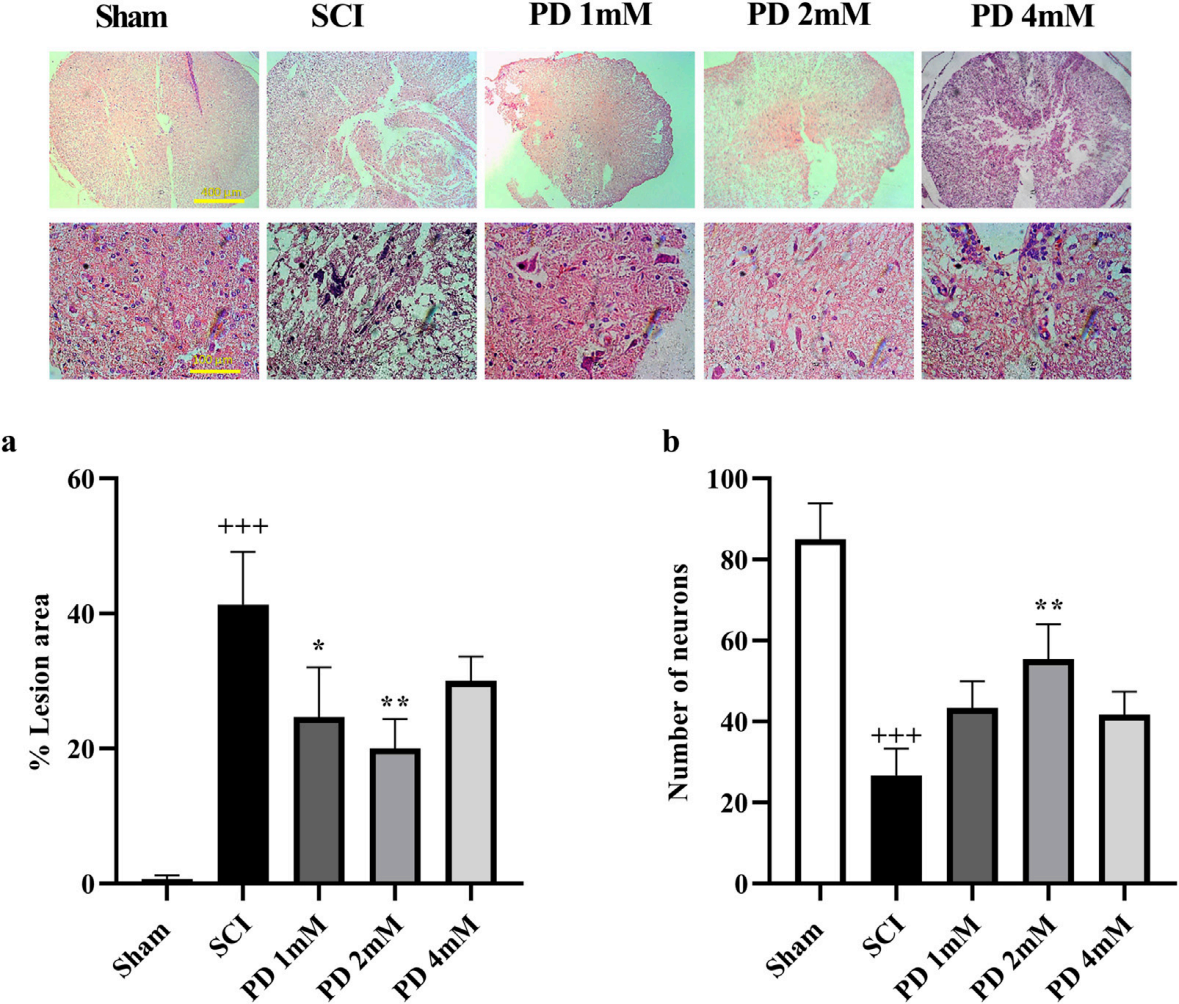
The impact of pelargonidin on the tissue change after SCI. Lesion area with 4X magnification **(a)** and the number of neurons with 40X magnification **(b)**. Data are expressed as mean ± SD. The statistical analysis was done using repeated measures of two-way ANOVA. ^+++^ *p* < 0.001 vs. sham group; ^*^ *p* < 0.05, ^**^ *p* < 0.01 vs. SCI group. SCI: spinal cord injury; PD: pelargonidin.

Following SCI, a significant amount of spinal cord tissue was damaged, with lesions occurring more frequently in the SCI group compared to the sham group (*p* < 0.001, Figure 6a). Histological analysis indicated specific characteristics of these lesions, including increased cavitation and neuronal loss. Treatment with pelargonidin, especially at a concentration of 2 mM, was associated with a reduction in both the severity and size of the lesions (*p* < 0.05).

Histopathological analysis demonstrated a substantial decrease of neurons in the spinal cord of the SCI group compared to the sham group (*p* < 0.001, Figure 6b). In contrast, the groups receiving pelargonidin, especially at the 2 mM dose, experienced a significant increase in spinal cord neuron counts (*p* < 0.01).

## 4 Discussion

The current study showed that pelargonidin significantly improved motor function and alleviated neuropathic pain (e.g., cold allodynia, heat hyperalgesia, and mechanical pain). Mechanistically, pelargonidin increased the level of glutathione and catalase, as well as MMP2 activity while reducing MMP9 activity and nitrite levels. Additionally, pelargonidin supports motor neuron survival in the spinal cord’s ventral horn, protecting against secondary damage from oxidative stress/inflammation. In almost the whole experiments of the current study, the dose of 2 mM shows better results than 1 mM and 4 mM. Such a method of dose-responsiveness refers to a reverse u-shaped dose-response effectiveness and the concept of hormesis (Peper, 2009; Fakhri et al., 2020; Fakhri et al., 2022c).

The initial trauma of SCI initiates a complex cascade of biological responses that further complicate recovery. One of the key consequences of SCI is the increase in ROS, which occurs as a result of disrupted cellular homeostasis. This elevation in ROS can be attributed to several factors, including mitochondrial dysfunction, inflammatory responses, and the activation of various enzymatic pathways, these processes contribute to oxidative stress and exacerbate neuronal damage (Yu et al., 2023). Elevated ROS levels cause oxidative stress by disrupting the balance between ROS production and detoxification (Yuan et al., 2021). Oxidative stress poses a significant threat to cellular wellbeing by causing damage to essential cellular components such as lipids, proteins, and DNA and can further intensify neuronal injury (Khatri et al., 2018; Singh et al., 2019). SCI initiates an inflammatory response that activates resident immune cells and recruits peripheral immune cells, releasing pro-inflammatory cytokines and chemokines that enhance oxidative stress and neuronal damage (Freyermuth-Trujillo et al., 2022). The relationship between oxidative stress and inflammation is bidirectional. On one hand, oxidative stress can activate signaling pathways that enhance the inflammatory response, while on the other hand, inflammatory mediators can increase ROS production. The combined effects of oxidative stress and inflammation can lead to programmed cell death, or apoptosis, in neurons. This process is characterized by specific morphological and biochemical changes, ultimately resulting in neuronal loss (Zhang et al., 2012; Behl et al., 2021). These changes and loss can lead to impaired motor and sensory functions, as well as other complications associated with SCI (Anjum et al., 2020).

Understanding and targeting these mechanisms is crucial for developing potential therapeutic strategies aimed at mitigating oxidative stress and inflammation, promoting neuronal survival, and enhancing recovery after SCIs. In this study, we provided evidence for the neuroprotective effects of intrathecal injection of pelargonidin administered 30 min after SCI. Intrathecal delivery is commonly used in SCI research as it allows for the direct delivery of therapeutic agents into the cerebrospinal fluid (CSF) and removes pharmacokinetic limitations. This method ensures higher localized concentrations of the drug at the site of injury while reducing systemic side effects. Employing such a methodology of intrathecal administration paves the road for the identification of the real efficacy of therapeutic agents (without pharmacokinetic limitations) towards developing novel formulations and other routes of administration. Intrathecal drug administration is also particularly beneficial for compounds that have difficulty crossing the blood-brain barrier or require rapid action within the central nervous system (Vaquero et al., 2018; Hu et al., 2023). The choice of administering pelargonidin 30 min’ post-injury is based on the understanding of the pathophysiological processes occurring immediately after SCI. The initial phase of injury involves primary mechanical damage, followed by a secondary phase characterized by inflammatory responses, oxidative stress, and excitotoxicity, which can exacerbate neuronal damage. Early intervention during this critical window aims to mitigate these secondary injuries, potentially improving recovery outcomes (Anjum et al., 2020; Jeong et al., 2021). Research indicates that interventions initiated shortly after injury can significantly influence long-term functional recovery (Zu et al., 2014).

Pelargonidin, 3,5,7-trihydroxy-2-(4-hydroxyphenyl)-1-benzopyrylium, is part of a larger group of secondary metabolites known as anthocyanins, which are widely recognized for their antioxidant properties and potential health benefits (Li et al., 2022). In the current study, we have shown that pelargonidin plays both direct (suppressing serum nitrite) and indirect (increasing antioxidant agents, catalase, and glutathione) effects on the modulation of oxidative stress. Previous reports also showed that pelargonidin functions as a potent antioxidant by scavenging free radicals and reducing oxidative damage at the cellular level. Numerous studies supported this property, demonstrating its capacity to neutralize ROS, which effectively lowers oxidative stress within cells (Sharath Babu et al., 2017; Xu et al., 2018; Li et al., 2022). Beyond its direct antioxidant actions, pelargonidin has the potential to bolster the body’s natural antioxidant defenses by increasing the expression of endogenous antioxidant enzymes. A key player in this process is the Keap1/nuclear factor erythroid 2-related factor 2 (Nrf2) signaling pathway, which mediates cellular responses to oxidative stress. By activating this pathway, pelargonidin facilitated the upregulation of protective enzymes, thereby enhancing overall cellular health. Research has shown that pelargonidin modulates gene expression within the Keap1/Nrf2 pathway and effectively mitigates citrinin-induced oxidative stress in HepG2 cells, highlighting its therapeutic potential in combating oxidative damage (Sharath Babu et al., 2017). This adaptive response can mitigate secondary injury in tissues affected by oxidative stress, thereby contributing to overall recovery. Our findings confirmed that pelargonidin indirectly modulates oxidative stress by acting as an antioxidant, as evidenced by the restoration of key antioxidant levels (glutathione and catalase) in the serum of SCI rats. Additionally, the observed decrease in serum nitrite levels after pelargonidin treatment reinforces its involvement in modulating oxidative stress. This is particularly noteworthy given the role of oxidative stress in exacerbating neuronal damage following SCI.

The neuroprotective effects of pelargonidin pass through a multifaceted approach that encompasses direct modulation of oxidative stress, reduction of inflammatory responses, and support for neuronal regeneration which all are interconnected. We have also previously shown a near interconnection between inflammation, oxidative stress, and apoptosis. This means that suppressing inflammation and oxidative stress could affect the apoptotic signaling pathways toward neuroprotection and neuronal regeneration (Fakhri et al., 2022a; Fakhri et al., 2022b). In line with our report, pelargonidin previously exhibited notable anti-inflammatory properties (Soleimani Asl et al., 2019; Henriques et al., 2020). MMP9 is often associated with increased inflammation and tissue damage, while MMP2 is linked to tissue remodeling and repair (Gravandi et al., 2024). Mechanical stimuli, ROS, and inflammatory mediators such as tumor necrosis factor-alpha can directly elevate MMP expression. MMP2 and MMP9 contribute to the breakdown of the blood-spinal cord barrier, increasing oxidative stress, demyelination, leukocyte trafficking, edema, and hemorrhage (Noble et al., 2002; Zhang et al., 2011). In injured tissue, MMP2 and MMP9 play a role in the inflammatory response by modulating the development of neuropathic pain. MMP9 is associated with microglia activation, while MMP2 is related to astrocyte activation (Schomberg et al., 2012). Our findings indicated that treatment with pelargonidin resulted in a notable modulation of MMPs, particularly the reduction of MMP9 and the restoration of MMP2 levels. The favorable shift in the balance of these MMPs suggested that pelargonidin may reduce the inflammatory response, thereby mitigating secondary injury processes that could hinder recovery.

Furthermore, a key finding of this study is the notable improvement in motor function and reduction of neuropathic pain in rats treated with pelargonidin. Supporting this, a study involving middle cerebral artery occlusion (MCAO) demonstrated that treatment with pelargonidin significantly improved neurological functions, as evidenced by reduced neurological severity scores compared to untreated MCAO rats (Fu et al., 2021). In models of diabetic neuropathy, pelargonidin treatment also led to a significant decrease in hyperalgesia, indicating its potential effectiveness in alleviating neuropathic pain associated with diabetes. Specifically, diabetic rats treated with pelargonidin showed improvements in both chemical and thermal hyperalgesia, which were linked to the compound’s antioxidant and anti-inflammatory properties, further underscoring its neuroprotective effects (Mirshekar et al., 2010). Also, studies have highlighted pelargonidin’s role in mitigating oxidative stress by reducing lipid peroxidation and enhancing the efficacy of endogenous antioxidant enzymes such as superoxide dismutase and catalase (Kowalczyk et al., 2024). *In vitro* studies have demonstrated that pelargonidin can inhibit the activation of nuclear factor kappa B (NF-κB), a key transcription factor involved in the inflammatory response. By modulating this pathway, pelargonidin can help reduce the expression of pro-inflammatory genes and enzymes such as inducible nitric oxide synthase (iNOS) and cyclooxygenase-2 (COX-2) (Kowalczyk et al., 2024; Scarpa et al., 2024). On the other hand, in studies involving 6-hydroxydopamine (6-OHDA)-induced toxicity, pelargonidin administration was effective in preserving dopaminergic neurons and improving behavioral outcomes in rats (Roghani et al., 2010). Our findings also were aligned with the neuroprotective effects of pelargonidin. The positive correlation between pelargonidin treatment and neuronal count highlights the compound’s neuroprotective properties.

Weight loss following SCI is indicative of metabolic dysfunction and can complicate recovery (Gorgey et al., 2014). The notable weight recovery observed in pelargonidin-treated rats further emphasizes the compound’s positive influence on overall health post-SCI. In a model of metabolic syndrome induced by a high-fat diet, pelargonidin 3-glucoside (P3G) enriched strawberries were shown to reduce abdominal fat and body weight gain in rats. This effect was associated with improvements in cardiovascular and liver health, suggesting that pelargonidin can positively influence body composition and metabolic parameters (Ghattamaneni et al., 2020). Moreover, a separate study indicated that daily administration of pelargonidin to mice over a month did not result in weight loss compared to control groups. This finding suggests that pelargonidin may not negatively impact body weight during treatment, highlighting its potential safety for regular use without adverse effects on weight recovery (Lamson et al., 2022). In previous studies, we found that compounds with antioxidant properties can effectively promote weight recovery in rats after SCI (Fakhri et al., 2019; Fakhri et al., 2021).

At the level of histopathological results, we showed herein that SCI made neuronal loss in ventral horn of spinal cord, which are responsible for motor activity. In the current report, we also confirmed that SCI caused demyelination of spinal cord. We further showed that pelargonidin improved compression SCI-induced degeneration by increasing motor neuron survival in ventral horn, while decreasing lesion size of spinal cord.

## 5 Conclusion

In conclusion, pelargonidin offers notable advantages in various areas, such as motor function recovery, pain relief, and metabolic health, through a multifaceted approach that includes direct/indirect modulation of oxidative stress, reduction of inflammation, and promotion of neuronal regeneration. Further research is crucial to fully understand the specific mechanisms and highlights the need for more rigorous mechanistic studies (e.g., apoptotic and neurogenesis pathways) by which pelargonidin exerts its beneficial effects in the context of SCI. Evaluating the effects of multiple doses of pelargonidin and in different routes of administration for a long period (e.g., 56–84 days) would be also valuable in future works.

## Data Availability

The raw data supporting the conclusions of this article will be made available by the authors, without undue reservation.
